# All-on-Six Implant-Supported Full-Arch Maxillary Rehabilitation With Indirect Sinus Elevation and Multi-unit Abutment Conversion: A Case Report

**DOI:** 10.7759/cureus.106800

**Published:** 2026-04-10

**Authors:** Sumit Aggarwal, Tarannum Jindal, Sauharad Sachdeva

**Affiliations:** 1 Prosthodontics And Crown &amp; Bridge, Subharti Dental College, Meerut, IND; 2 Prosthodontics, Subharti Dental College, Meerut, IND

**Keywords:** all-on-six concept, full-arch maxillary rehabilitation, indirect sinus lift, multi-unit abutments, screw-retained implant prosthesis

## Abstract

Full-arch implant-supported rehabilitation of the edentulous maxilla presents significant surgical and prosthetic challenges, particularly in achieving biomechanical stability and optimal esthetics. This case report describes prosthetically driven full-arch rehabilitation of a 33-year-old female patient using the All-on-Six concept in the maxillary arch. Following comprehensive clinical and Cone Beam Computed Tomography (CBCT) evaluation, six implants were placed freehand in regions #13, #14, #16, #23, #24, and #26. An indirect sinus lift was performed in the #26 region due to limited residual bone height (8 mm). The primary stability during implant placement was between 30-50 Ncm, and the Implant Stability Quotient (ISQ) value was 65; therefore, a delayed loading protocol was adopted. After a five-month healing period, multi-unit abutments (0°, 17°, and 30°) were placed to achieve prosthetic parallelism. A splinted open-tray impression technique was used, and a digital workflow with Exocad software facilitated framework design. A screw-retained porcelain-fused-to-metal (PFM) prosthesis was delivered and torqued to 35 Ncm. Mandibular rehabilitation included crown lengthening and PFM crowns. Postoperative radiographic evaluation confirmed satisfactory implant positioning and prosthetic adaptation. This case demonstrated that meticulous surgical planning, prosthetic precision, and evidence-based protocols contribute to predictable functional and esthetic outcomes in full-arch maxillary rehabilitation.

## Introduction

Implant-supported full-arch fixed dental prostheses are widely used as a predictable alternative to conventional complete dentures in edentulous patients, offering enhanced function, stability, and improved patient satisfaction. Implant-supported full-arch rehabilitation of the maxilla was historically considered more challenging than its mandibular counterpart due to the comparatively lower bone density of the maxillary arch [1].

In 2003, the All-on-Four treatment concept was introduced by Malo et al. to support full-arch fixed dental prostheses using four implants. Although the All-on-Four concept is more widely known and formally branded, the All-on-Six approach evolved as a modification, utilizing six implants to enhance load distribution, biomechanical stability, and long-term prognosis, particularly in cases with adequate bone volume [2].

Furthermore, the All-on-Six concept offers several advantages, including increased bone anchorage, reduced cantilever length, and a decreased risk of damage to anatomical structures through the strategic use of angled implant placement. This treatment concept is used to rehabilitate completely edentulous maxillary arches with a fixed dental prosthesis supported by six implants. Typically, two implants are placed axially in the anterior region, two implants are positioned in the premolar region, and two implants are placed in the posterior region, often with distal angulation to maximize anterior-posterior spread and reduce cantilever length [3].

This configuration enhances biomechanical load distribution, improves primary stability in the maxilla, and provides increased prosthetic support for full-arch rehabilitation. Successful outcomes with this approach depend on careful case selection. Therefore, the surgical protocol should be thoroughly assessed through radiographic and clinical evaluations, combined with meticulous prosthetic planning, prior to implementation. Functional considerations, such as occlusal relationships and prosthetic stability, should also be evaluated to ensure that the treatment provides not only esthetic satisfaction but also optimal functional outcomes for the patient [4].

The purpose of this study is to report a case of full-arch rehabilitation of the maxilla using six endosseous implants, followed by prosthetic rehabilitation according to established clinical protocols.

## Case presentation

**Figure 1:**
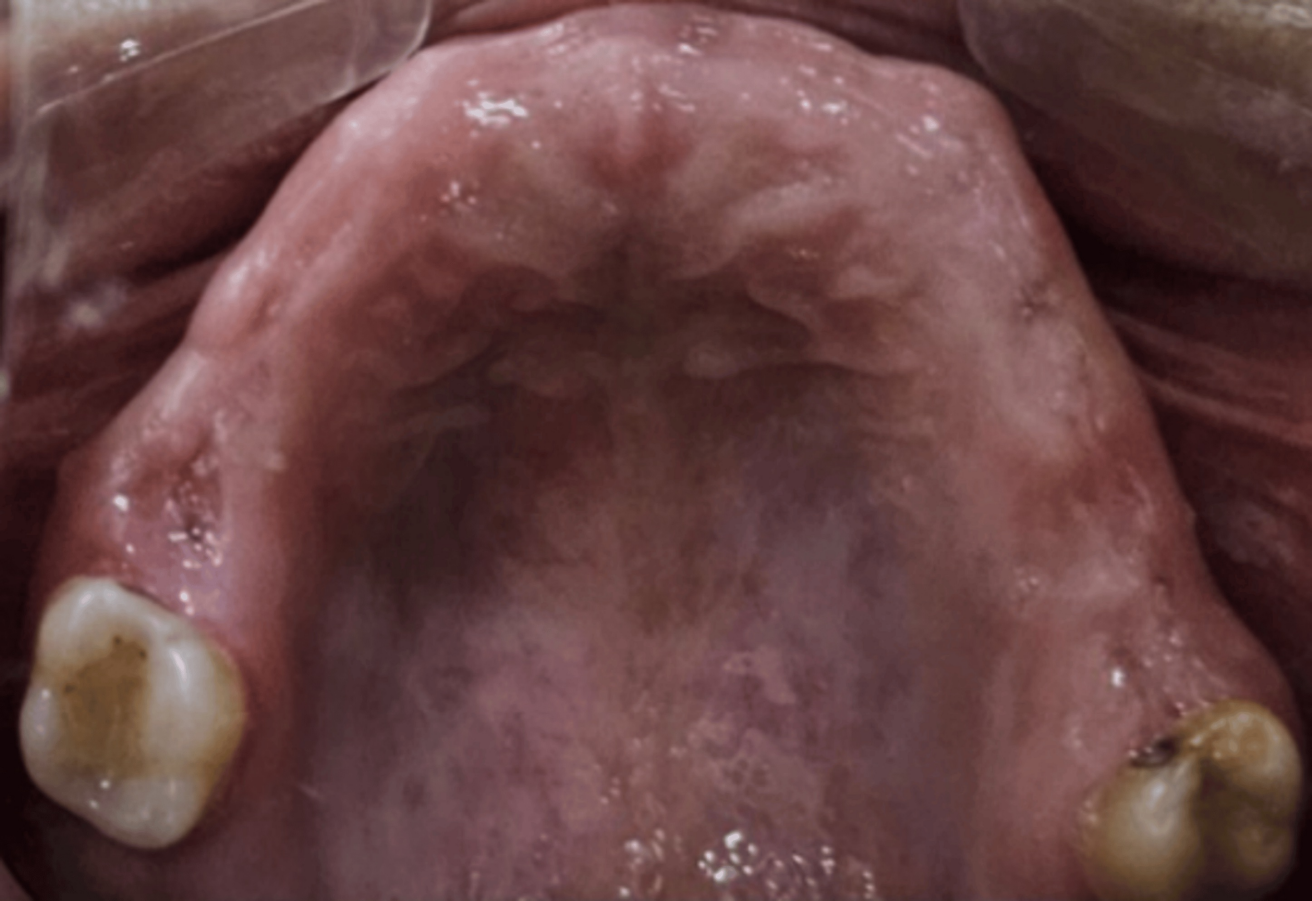
Intraoral view of maxillary arch Intraoral view exhibiting partial edentulism in maxillary arch.

**Figure 2:**
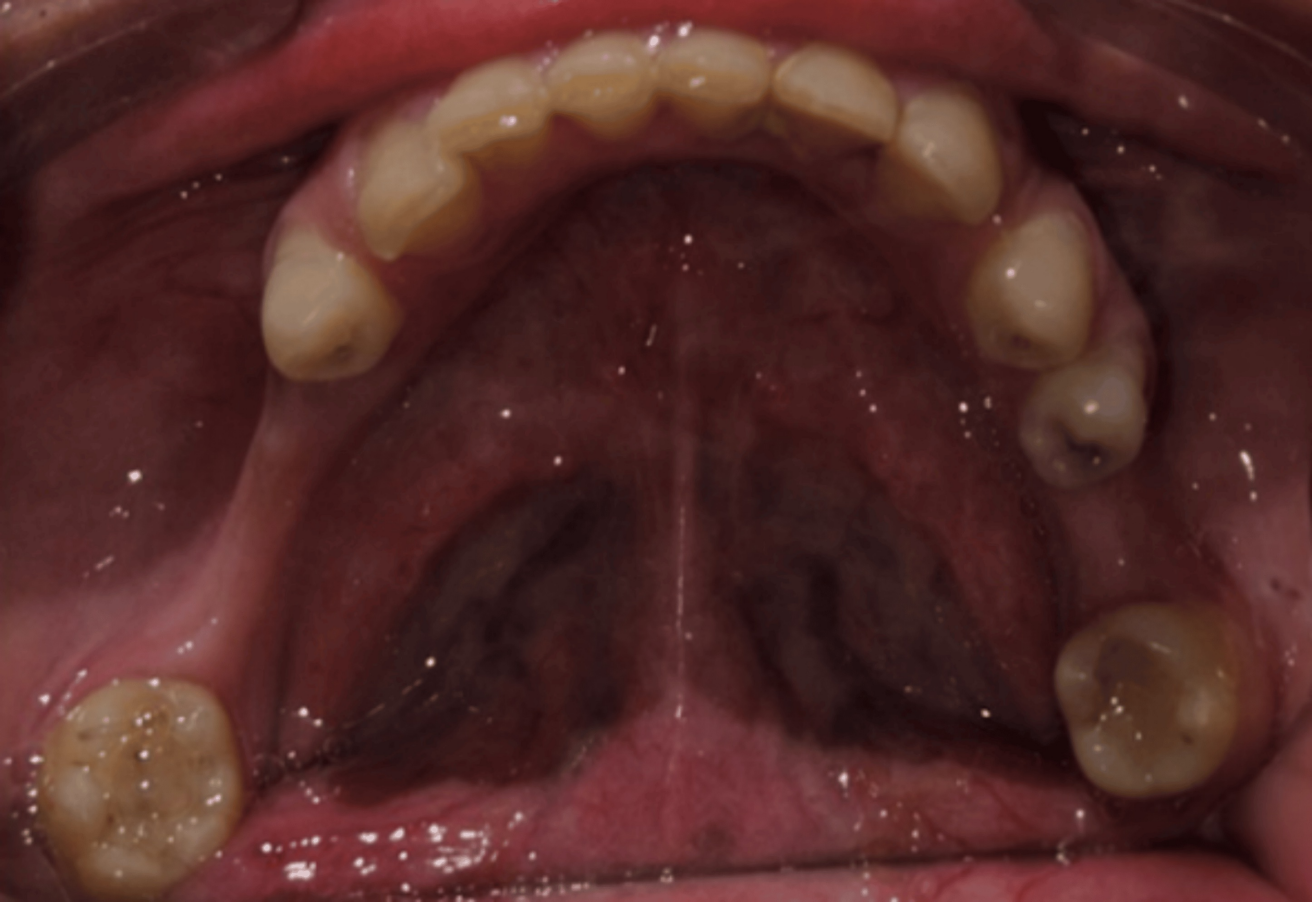
Intraoral view of the mandible Intraoral view showing partial edentulism in the mandible.

A 33-year-old female patient reported to the Department of Prosthodontics And Crown & Bridge with a chief complaint of missing teeth in the maxillary and mandibular arches. On intraoral examination, the maxillary arch exhibited partial edentulism with missing teeth at positions 11, 21, 13, 14, 15, 16, 21, 22, 23, 24, 25, and 26. In the mandibular arch, teeth at positions 35, 36, 37, and 46 were clinically absent (Figures 1, 2).

Surgical phase

A comprehensive preoperative evaluation was performed prior to implant placement. During extraction, the bone quality was clinically assessed, and it corresponded to D2-D3 bone density. Cone-beam computed tomography (CBCT) was used to evaluate alveolar bone height, width, and proximity to vital anatomical structures, particularly the maxillary sinus.

In the region of tooth #26, the residual bone height was approximately 8 mm, indicating limited vertical bone availability. Considering the proximity of the maxillary sinus, an indirect sinus lift procedure was planned to facilitate implant placement in this region. Careful treatment planning was undertaken to minimize the risk of sinus membrane perforation and to ensure adequate bone support for long-term implant stability.

Local anesthesia was administered using 2% lignocaine with 1:100,000 adrenaline via infiltration technique. A total of six implants were placed in relation to teeth #13, #14, #16, #23, #24, and #26. The implant dimensions were as follows: 1) #13: 3.3 × 13 mm; 2) #14: 3.75 × 13 mm; 3) #16: 5.0 × 10 mm; 4) #23: 3.3 × 13 mm; 5) #24: 3.3 × 13 mm; and 6) #26: 4.2 × 8 mm

**Figure 3:**
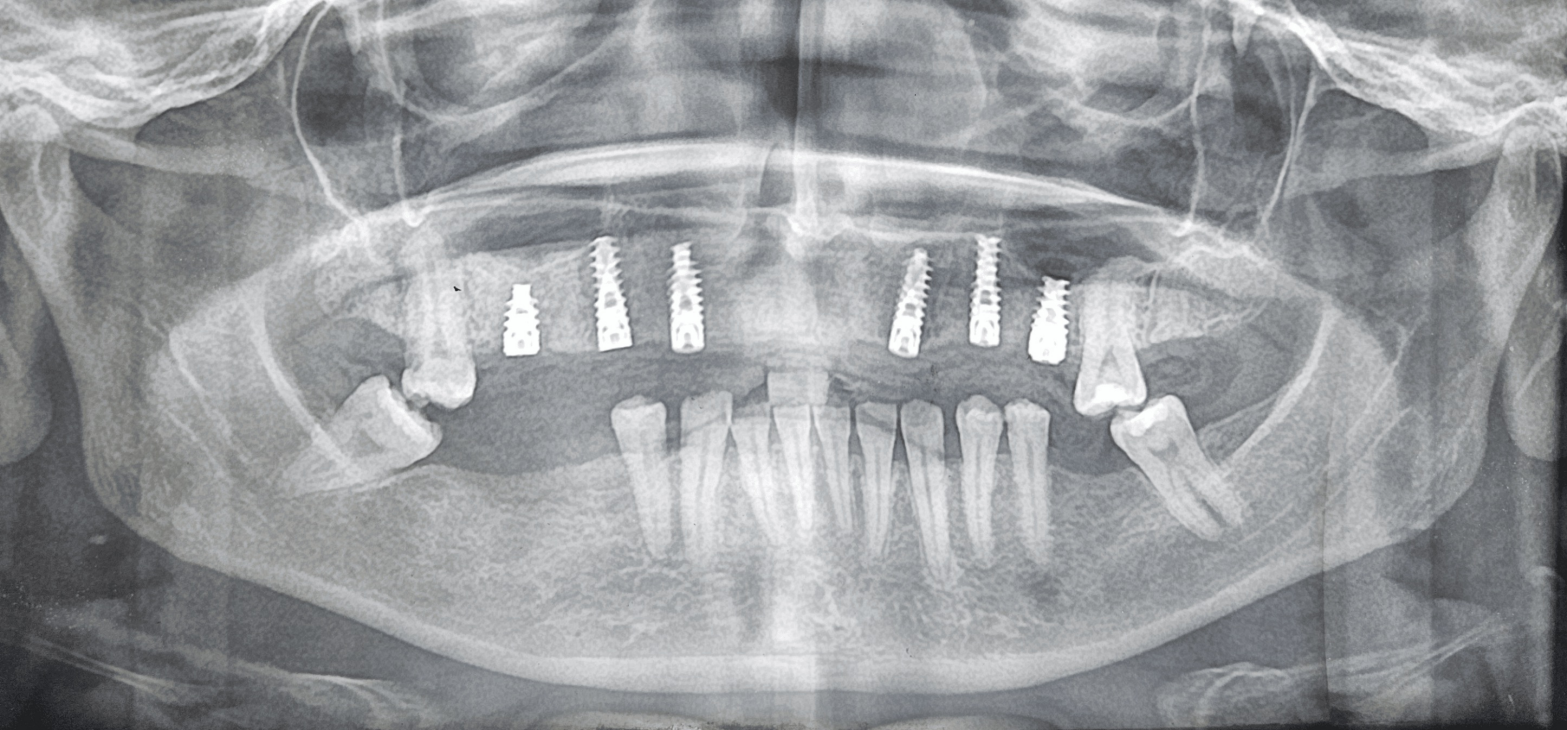
Postoperative orthopantomogram (OPG) OPG after implant placement.

The primary stability during implant placement was between 30-50 Ncm, and the Implant Stability Quotient (ISQ) value was 65. Given the moderate primary stability achieved, a delayed loading protocol was planned. Cover screws were placed, and all the implants were submerged to allow for undisturbed osseointegration (Figure 3).

A healing period of five months was observed.

**Figure 4:**
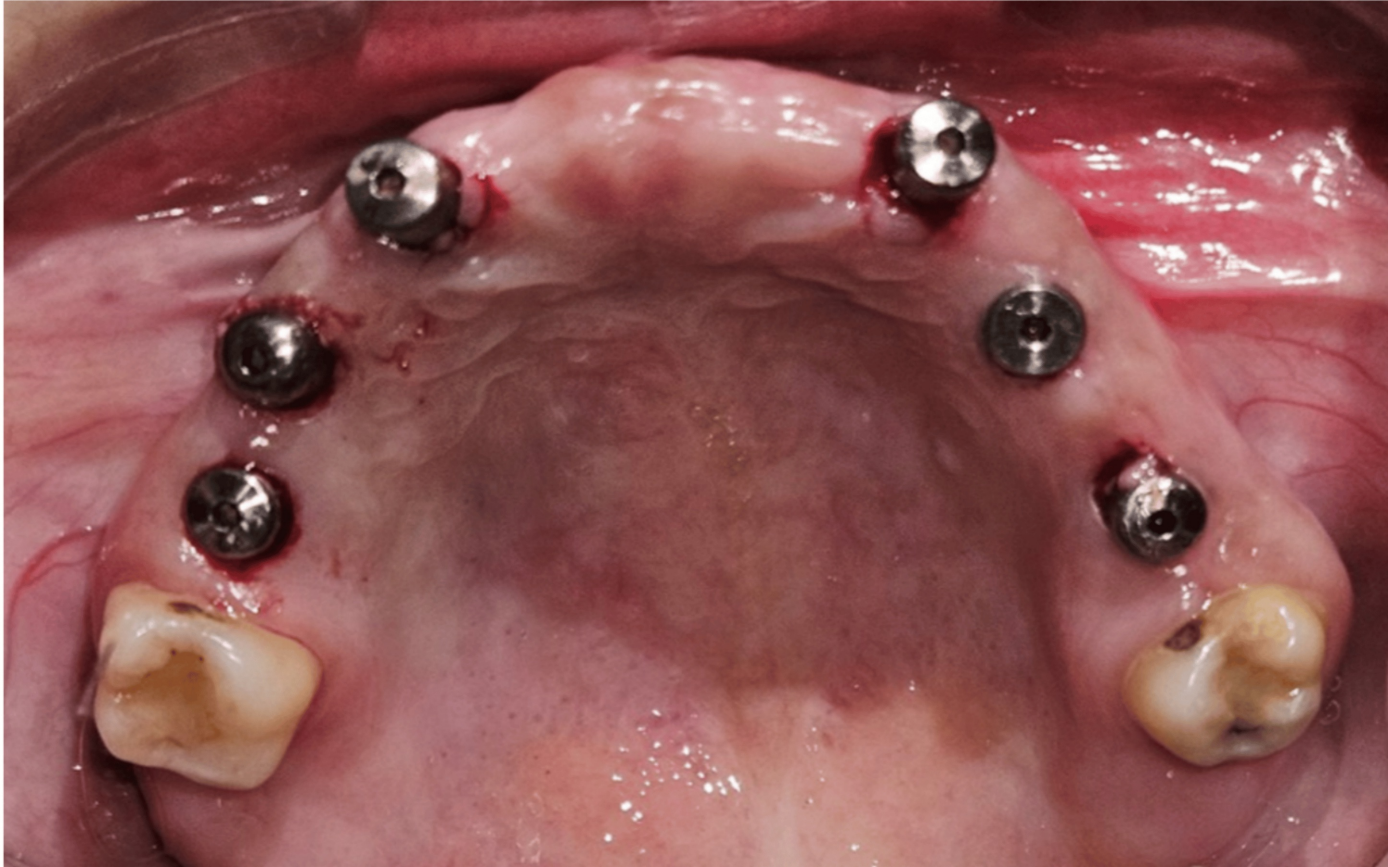
Intraoral view of the maxilla Intraoral view showing placement of healing abutments.

Following the osseointegration phase, a second-stage surgical procedure was performed. The implants were exposed, cover screws were removed, and healing abutments were placed. The healing abutments were maintained for approximately 15 days to facilitate soft tissue maturation and the formation of an adequate peri-implant gingival collar prior to prosthetic rehabilitation (Figure 4).

Prosthetic phase

Following completion of the soft tissue healing phase, the healing abutments were removed, and the peri-implant tissues were evaluated for adequate gingival collar formation. Multi-unit abutments (MUAs) were then selected based on implant angulation and prosthetic requirements to achieve a common path of insertion and facilitate screw-retained prosthetic rehabilitation.

**Figure 5:**
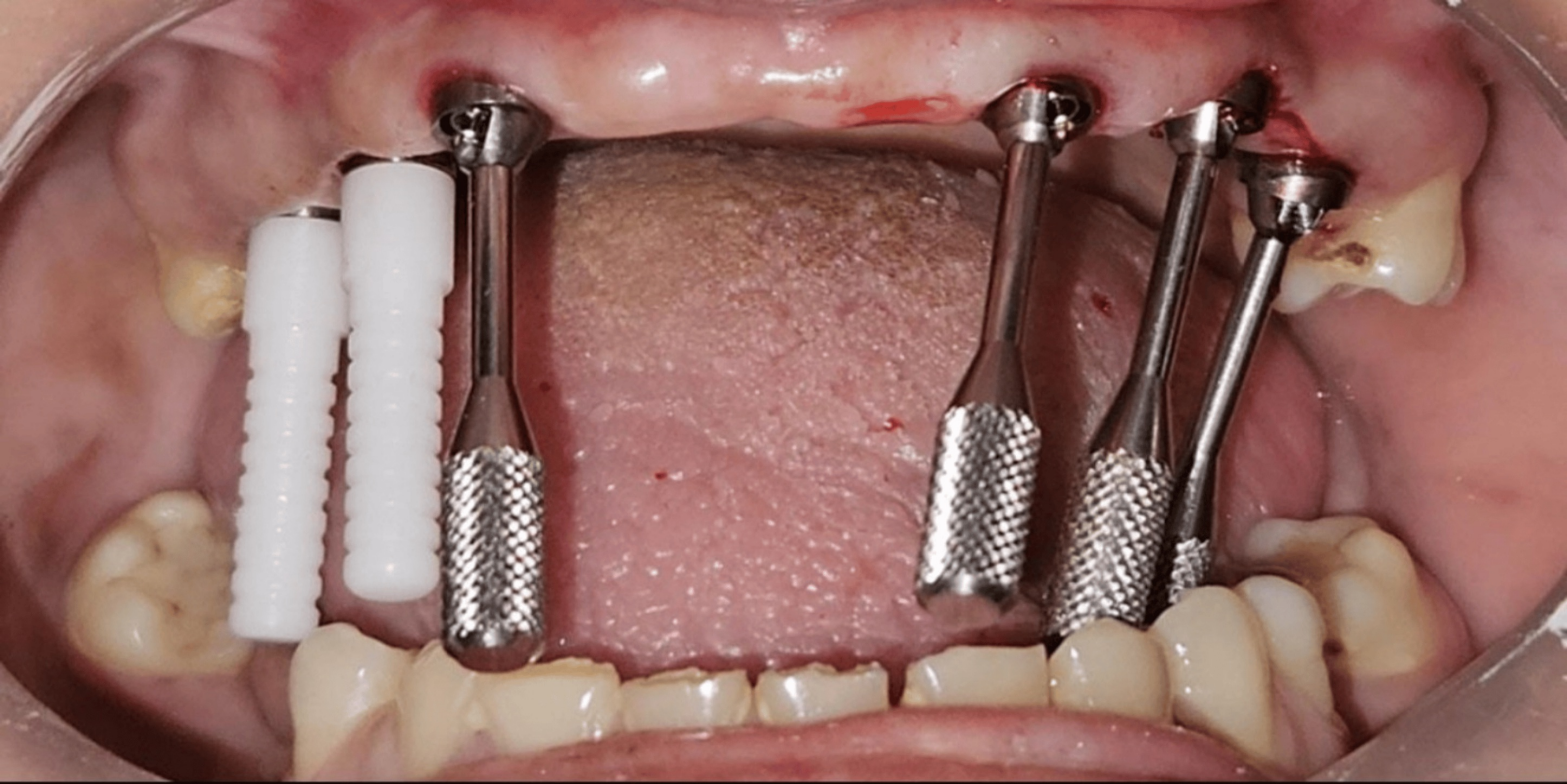
Intraoral view of the maxilla Intraoral view showing the placement of the six multi-unit abutments.

A 0-degree MUA was placed in relation to tooth #13. Seventeen-degree MUAs were placed in relation to teeth #14, #23, and #24 to compensate for minor implant angulation discrepancies. In the posterior regions, 30-degree MUAs were placed in relation to teeth #16 and #26 to correct greater implant angulation and establish prosthetic parallelism. All MUAs were secured according to the manufacturer’s recommended torque values (Figure 5).

**Figure 6:**
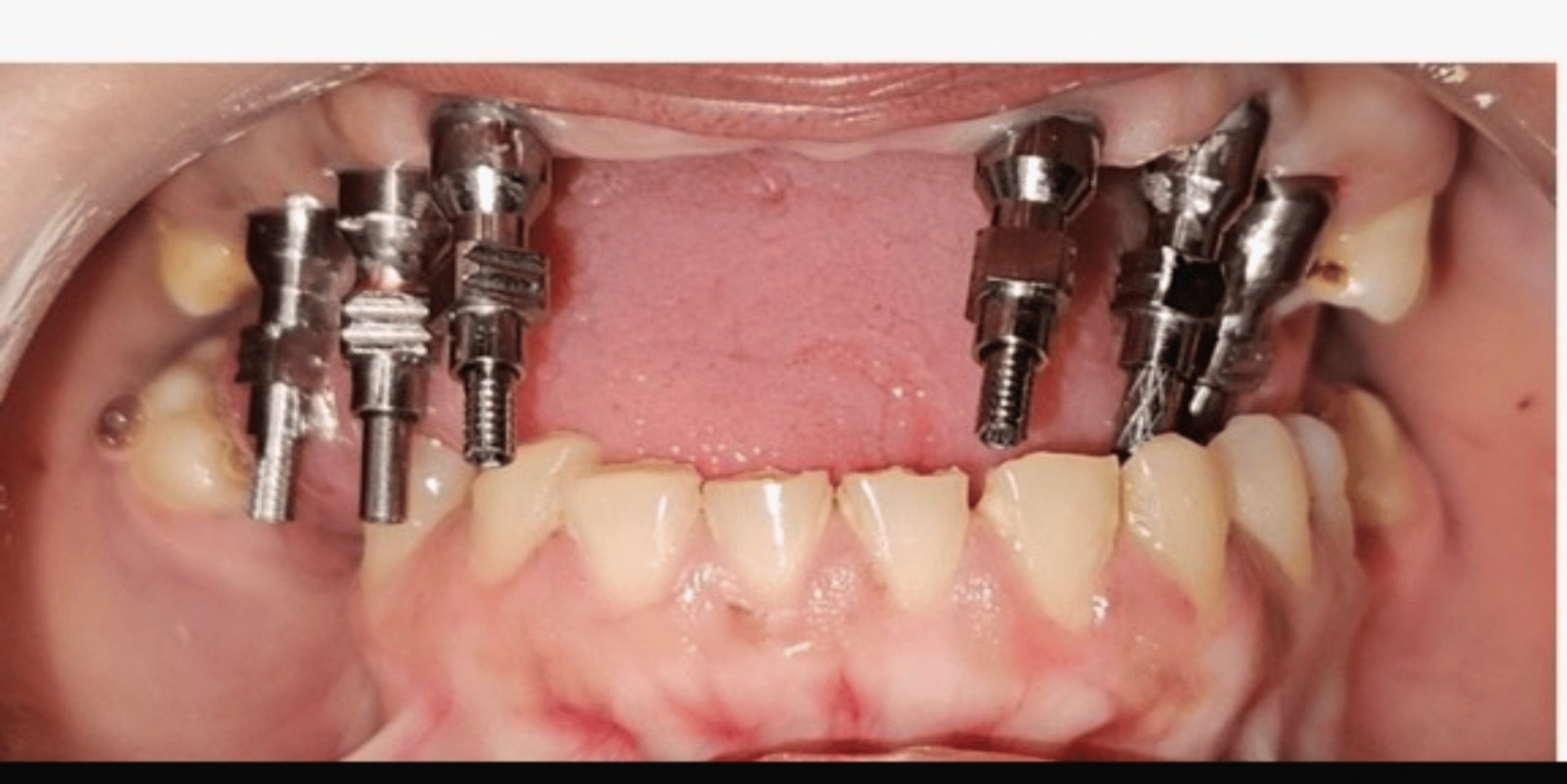
Placement of the six multi-unit impression copings Placement of six multi-unit open tray impression copings.

Following placement of the MUAs, open-tray impression copings compatible with the MUAs were selected and seated (Figure 6).

**Figure 7:**
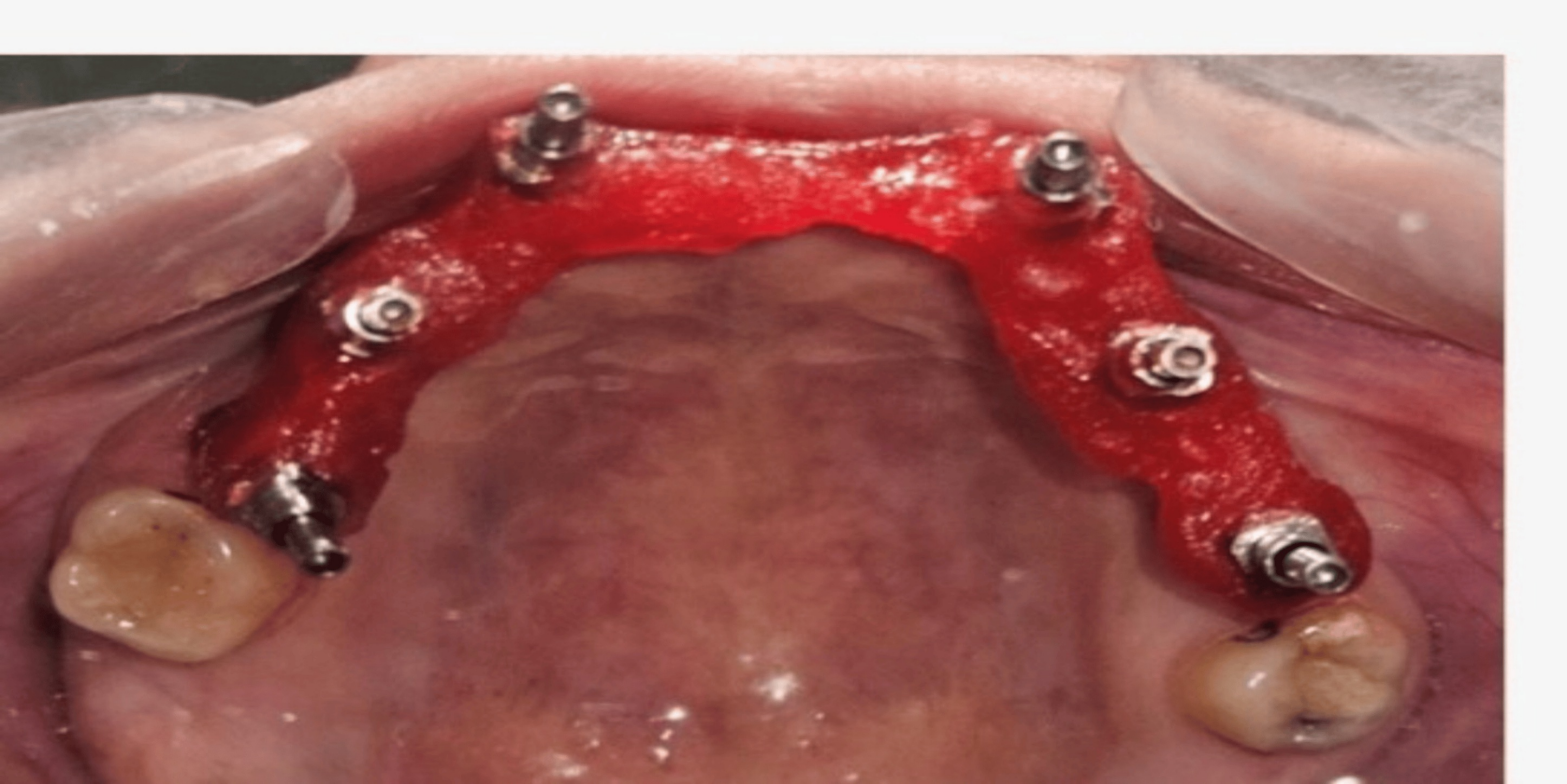
Splinted impression copings Copings were splinted intraorally using dental floss and autopolymerizing pattern resin.

Complete seating was verified clinically to ensure accurate engagement. To enhance impression accuracy and minimize micromovement, the impression copings were splinted intraorally using dental floss and autopolymerizing pattern resin, thereby creating a rigid assembly for precise transfer of implant positions (Figure 7).

**Figure 8:**
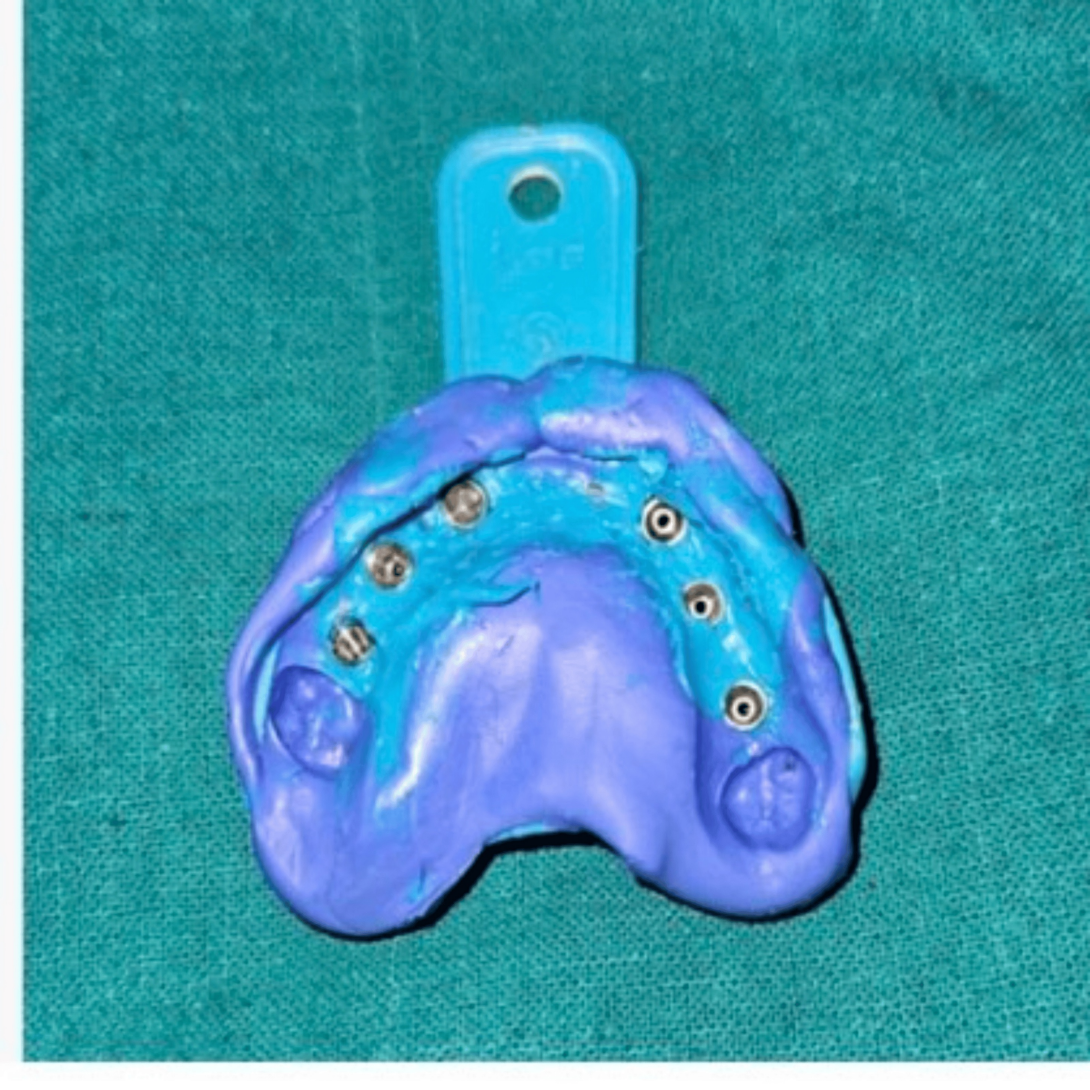
Final impression Final impression made using polyvinylsiloxane material

After polymerization of the splinting material, an open-tray impression was made using an elastomeric impression material. Once the material had set, the copings were unscrewed through the tray access openings, and the impression was carefully retrieved (Figure 8).

Multi-unit analogs were then attached to the impression copings within the impression, facilitating the fabrication of an accurate master cast for definitive prosthetic rehabilitation.

Following impression making and master cast fabrication, maxillomandibular relationship records were obtained. Wax occlusal rims were fabricated over the record bases, and the jaw relation was recorded. The occlusal vertical dimension (OVD) was carefully evaluated and verified clinically to establish appropriate facial proportions, phonetics, and interarch space. A face-bow transfer record was obtained to relate the maxillary cast to the hinge axis of the articulator, thereby facilitating accurate simulation of mandibular movements. The definitive casts were then mounted on a semi-adjustable articulator using the face-bow and centric relation records.

Subsequently, the mounted master cast was digitized using a laboratory scanner to initiate the digital workflow. The prosthesis was fabricated using a FastForm Direct Metal Laser Sintering (DMLS) machine (FastForm 3D Technology Co. Ltd., China) in conjunction with DentalCAD 3.0 software (Exocad GmbH, Germany), ensuring an appropriate emergence profile, passive fit, occlusal scheme, and prosthetic contours in accordance with functional and esthetic requirements.

**Figure 9:**
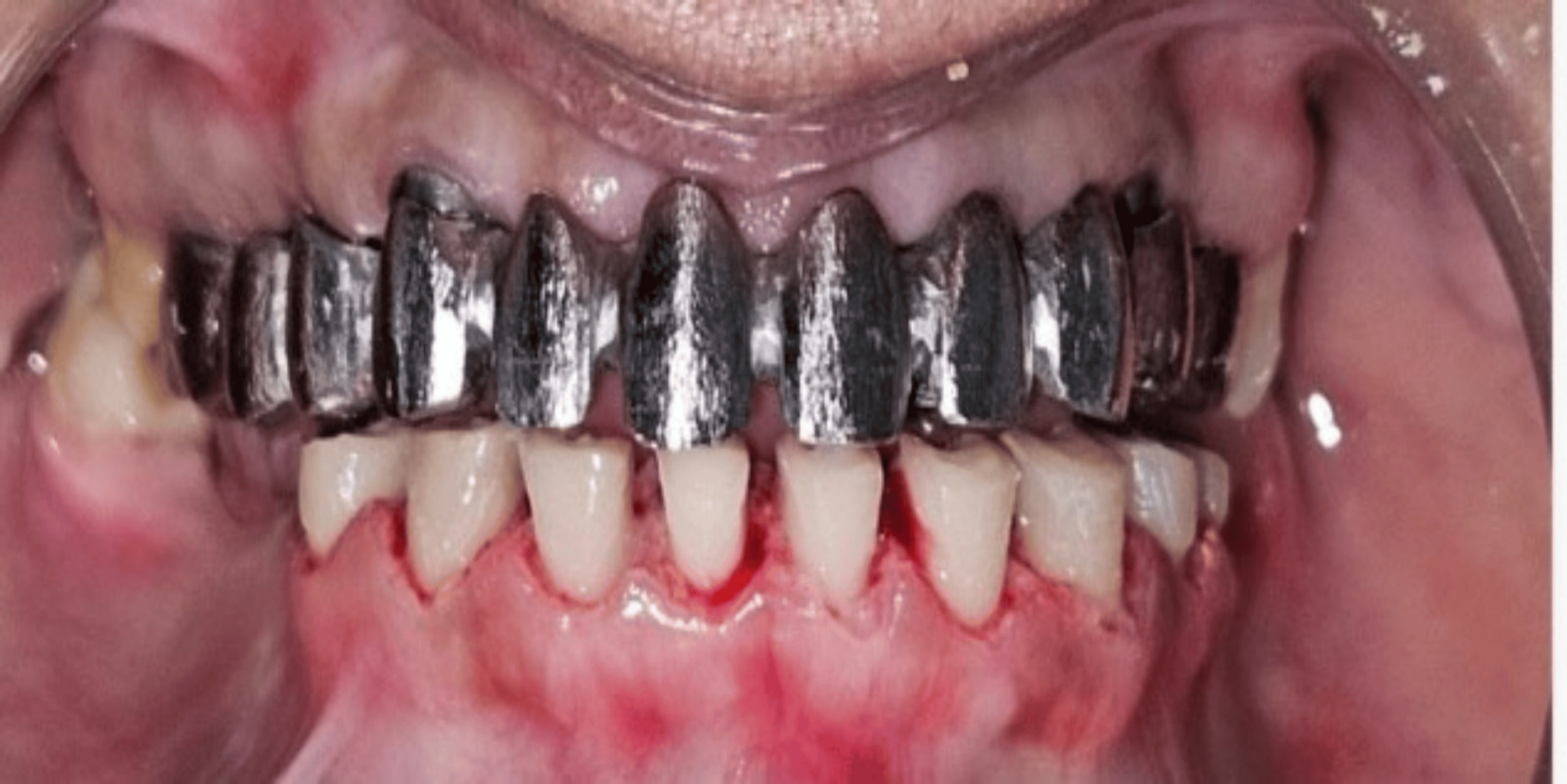
Metal try-in

In the maxillary arch, a screw-retained porcelain-fused-to-metal (PFM) implant-supported prosthesis was planned and fabricated following completion of the digital design and framework verification procedures. The PFM prosthesis was selected considering its favorable mechanical strength, long-term clinical reliability, and cost-effectiveness, particularly for full-arch rehabilitation. Try-in procedures were performed to evaluate passive fit, occlusion, esthetics, and phonetics prior to final prosthesis delivery (Figure 9).

The prosthetic screws were tightened to a torque of 35 Ncm in accordance with the manufacturer’s recommendations to ensure optimal preload and minimize the risk of screw loosening. The screw access openings were subsequently sealed, and occlusion was carefully refined to achieve harmonious centric and eccentric contacts.

**Figure 10:**
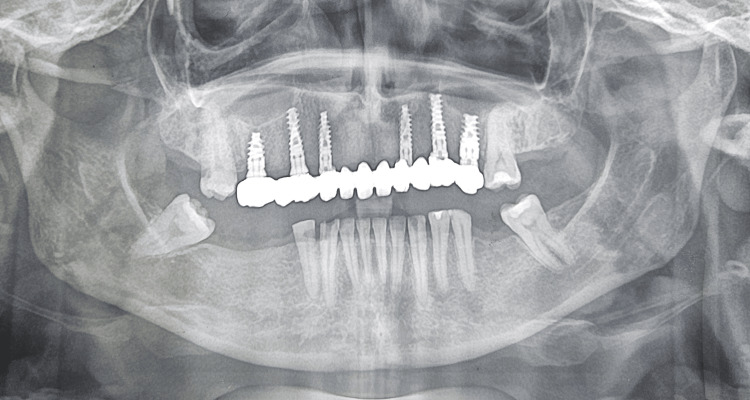
Postoperative orthopantomogram (OPG) Postoperative OPG evaluating the final positioning of the implants and prosthesis.

A postoperative orthopantomogram (OPG) was obtained to evaluate the final positioning of the implants and prosthesis, verify complete seating of the prosthetic components, and assess the peri-implant bone levels. The radiograph confirmed satisfactory implant alignment and prosthetic adaptation (Figure 10).

In the mandibular arch, a multidisciplinary restorative approach was undertaken. Clinical crown lengthening was performed in relation to teeth #31 to #35 and #41 to #44 to achieve adequate clinical crown height and enhance the long-term restorative prognosis. Following appropriate healing and periodontal stabilization, definitive tooth preparations were carried out in accordance with established biomechanical principles.

**Figure 11:**
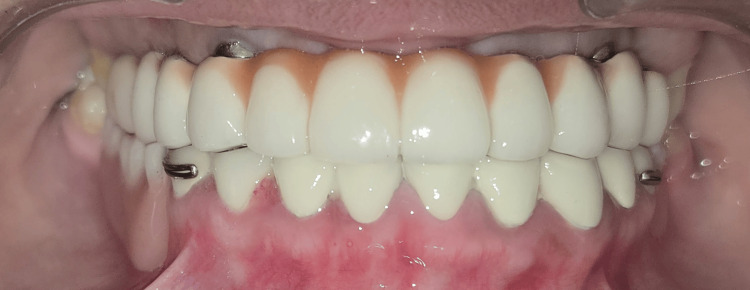
Postoperative intraoral view Postoperative intraoral view with porcelain-fused-to-metal (PFM) crowns on prepared mandibular teeth.

PFM crowns were fabricated for the prepared mandibular teeth. Prior to cementation, marginal adaptation, proximal contacts, occlusal harmony, and esthetic integration were meticulously evaluated. Due to financial constraints, the patient declined implant therapy in the posterior mandibular region. Therefore, the posterior edentulous area of the mandible was rehabilitated using a removable partial denture (RPD). The crowns were subsequently definitively cemented, and postoperative instructions were provided (Figure 11).

The patient was placed on a structured follow-up protocol for periodic evaluation of peri-implant and periodontal health.

## Discussion

Reconstruction of the edentulous maxilla, particularly when soft tissue esthetics are involved, represents a complex clinical challenge and often necessitates an interdisciplinary approach. The introduction of four to six endosseous implants in the anterior region of the edentulous maxilla and mandible to support a full-arch fixed prosthesis, as described by Brånemark et al., established the foundational principles of modern implant dentistry. These principles continue to guide contemporary full-arch rehabilitation protocols [5].

Long-term evidence supports the predictability of implant therapy performed using conventional techniques. Jung et al. (2012) demonstrated high long-term survival rates of freehand implant placement that are comparable to guided approaches in many clinical situations [6]. Similarly, Pjetursson et al. reported five-year survival rates of approximately 95-96% for fixed implant-supported prostheses, further reinforcing the reliability of implant-supported full-arch rehabilitations [7].

The present case illustrates a prosthetically driven full-arch rehabilitation of the maxilla utilizing the All-on-Six concept, followed by multi-unit abutment conversion, a splinted open-tray impression technique, digital CAD design using Exocad software, and delivery of a screw-retained PFM definitive prosthesis. Comparative literature evaluating the All-on-Four and All-on-Six concepts suggests that although the latter approach may involve increased cost and potentially longer treatment duration, it offers biomechanical advantages through the placement of additional implants and improved stress distribution [8].

The All-on-Six configuration reduces cantilever length and associated biomechanical complications. Research by Hassan et al. supports the biomechanical superiority of the All-on-Six concept and additionally highlights improved oral hygiene parameters, including lower plaque levels, when compared with All-on-Four restorations. These findings emphasize the importance of meticulous prosthetic planning and strict maintenance protocols to achieve optimal long-term outcomes [9].

In the present case, an indirect sinus lift was performed in the region of #26 due to limited residual bone height. According to Del Fabbro et al., osteotome-mediated sinus floor elevation (OMSFE) is a predictable and reliable technique for implant placement in the posterior maxilla when residual bone height is compromised. Implant survival rates following OMSFE are generally reported to be above 90% and are often comparable to implants placed in native bone, particularly when residual bone height is ≥5-6 mm [10].

According to Joda et al., the use of a digital CAD-CAM workflow employing Exocad software for the design and fabrication of implant-supported prostheses has gained widespread acceptance due to its improved precision, efficiency, and predictability in prosthetic outcomes [11]. When combined with screw-retained prosthetic designs, such digital workflows align with evidence-based protocols associated with favorable clinical outcomes. Pjetursson et al., in their systematic reviews, reported medium-term survival rates of implant-supported fixed dental prostheses ranging between approximately 90% and 97%, highlighting their high predictability when appropriate surgical and prosthetic protocols are followed [12].

In the present rehabilitation, the prosthesis was torqued to 35 Ncm in accordance with commonly recommended manufacturer guidelines for implant prosthetic screws, which helps minimize screw loosening and maintains prosthetic stability [13]. MUAs are commonly employed in full-arch implant rehabilitations to correct implant angulation and establish a favorable prosthetic path of insertion. Their use improves prosthetic alignment and facilitates the fabrication of screw-retained restorations, particularly in cases involving tilted implants. 

Splinted open-tray impression techniques are widely recommended in cases involving multiple implants due to their superior accuracy in transferring implant positions compared with unsplinted methods. Previous studies have demonstrated that splinting impression copings reduces transfer distortion and improves the likelihood of obtaining a passive-fitting prosthetic framework, thereby minimizing prosthetic complications that may compromise long-term implant success [14]. Achieving a passive fit remains critical in implant prosthodontics because misfit between the framework and implant components can lead to mechanical complications such as screw loosening, component fracture, or marginal bone loss [13].

When combined with precise surgical placement, appropriate loading protocols, and regular maintenance, prosthetic accuracy significantly contributes to favorable implant and prosthesis survival outcomes. Within the limitations of this case report, prosthetically driven full-arch rehabilitation of the edentulous maxilla using the All-on-Six concept demonstrated predictable clinical outcomes when supported by comprehensive CBCT-based treatment planning, accurate surgical execution, and a structured prosthetic workflow. The use of MUAs for angulation correction, splinted open-tray impressions to enhance transfer accuracy, and digital CAD/CAM design protocols contributed to improved prosthetic fit and biomechanical stability.

In addition, the adjunctive indirect sinus lift procedure performed in the posterior maxilla facilitated implant placement in regions with reduced residual bone height. Minimally invasive sinus elevation techniques have been reported to achieve high implant survival rates and predictable bone augmentation outcomes, making them a reliable approach in posterior maxillary implant rehabilitation [15].

Overall, adherence to evidence-based principles, careful loading protocols, and meticulous prosthetic planning remain fundamental determinants of long-term implant success. Regular follow-up and maintenance are essential to preserve peri-implant health and prosthetic integrity. This case reinforces that, when executed with comprehensive interdisciplinary planning and precise clinical implementation, full-arch implant rehabilitation can achieve high survival rates and satisfactory functional and esthetic outcomes.

## Conclusions

Full-arch rehabilitation of the edentulous maxilla using the All-on-Six concept offers a stable and biomechanically favorable treatment modality when performed with comprehensive planning and precise clinical protocols. In the present case, the integration of CBCT-based assessment, strategic implant positioning, indirect sinus elevation, and angulation correction through MUAs enabled restoration of function and esthetics in a predictable manner.

The combination of conventional surgical principles with a structured prosthetic workflow and digital design facilitated accurate framework fabrication and optimal load distribution. Careful loading decisions and appropriate torque control further enhanced prosthetic stability. This case highlights that individualized treatment planning, sound biomechanical considerations, and long-term maintenance protocols are essential for achieving durable outcomes in complex maxillary full-arch implant rehabilitations.
